# Nitrogen-Doped Graphene Monolith Catalysts for Oxidative Dehydrogenation of Propane

**DOI:** 10.3389/fchem.2021.759936

**Published:** 2021-10-15

**Authors:** Weijie Liu, Tianlong Cao, Xueya Dai, Yunli Bai, Xingyu Lu, Fan Li, Wei Qi

**Affiliations:** ^1^ Shenyang National Laboratory for Materials Science, Institute of Metal Research, Chinese Academy of Sciences, Shenyang, China; ^2^ School of Materials Science and Engineering, University of Science and Technology of China, Shenyang, China

**Keywords:** nitrogen doping, graphene monolith, oxidative dehydrogenation of propane, nanocarbon materials, kinetic analysis

## Abstract

It’s of paramount importance to develop renewable nanocarbon materials to replace conventional precious metal catalysts in alkane dehydrogenation reactions. Graphene-based materials with high surface area have great potential for light alkane dehydrogenation. However, the powder-like state of the graphene-based materials seriously limits their potential industrial applications. In the present work, a new synthetic route is designed to fabricate nitrogen-doped graphene-based monolith catalysts for oxidative dehydrogenation of propane. The synthetic strategy combines the hydrothermal-aerogel and the post thermo-treatment procedures with urea and graphene as precursors. The structural characterization and kinetic analysis show that the monolithic catalyst well maintains the structural advantages of graphene with relatively high surface area and excellent thermal stability. The homogeneous distributed nitrogen species can effectively improve the yield of propylene (5.3% vs. 1.9%) and lower the activation energy (62.6 kJ mol^−1^ vs. 80.1 kJ mol^−1^) in oxidative dehydrogenation of propane reaction comparing with un-doped graphene monolith. An optimized doping amount at 1:1 weight content of the graphene to urea precursors could exhibit the best catalytic performance. The present work paves the way for developing novel and efficient nitrogen-doped graphene monolithic catalysts for oxidative dehydrogenation reactions of propane.

## Introduction

Propylene is one of the most important industrial building blocks, which can be used extensively to produce a variety of high value-added petrochemical products. (Sattler et al., 2014). The oxidative dehydrogenation of propane (ODH) is a very promising route to produce propylene, because it is an exothermic reaction without reaction equilibrium limitation (Carrero et al., 2014). The most serious problems of the present industrial catalysts for this reaction system are the relatively low selectivity of propylene and poor stability of the catalysts due to the occurrence of deep oxidation in the oxygen atmosphere at relatively high temperature (Xiong et al., 2019). Recently, nanocarbon materials (Lin and Su, 2014; Qi et al., 2015; Roldan et al., 2015) are playing an important role in dehydrogenation reactions including the dehydrogenation of ethylbenzene, oxidative dehydrogenation of propane and butane etc. due to their advantages in adjustable surface acid/alkaline property, sustainability, environment friendly and low cost (Yan P. et al., 2019; Zhang et al., 2020). However, nanocarbon materials are usually in the form of powder, which seriously limits their industrial applications in gas-phase heterogeneous catalysis due to the obvious pressure drop in the fixed-bed reactor and the difficulty in mass and heat transfer (Garcia-Bordeje et al., 2006; Liu et al., 2014). More seriously, the powder form catalysts may trigger a series of safety issues, such as the danger of explosion etc. The intense friction and contraction between powder nanomaterials will also result in significant changes in the surface properties during the long-term operation cycle, accounting for the deactivation in some degree. On the other hand, the nanocarbon materials usually have a decent catalytic activity with surface defects and oxygen-containing functional groups as catalytic activity sites comparing with the conventional metal-based industrial catalysts. It is also a great scientific challenge to improve the catalytic performance of nanocarbon catalysts *via* controllable synthesis/modification strategies.

In order to achieve the potential of the powder form nanocarbon catalysts in practical industrial applications, it is necessary to transform the powder into granule or monolith materials, especially for carbon materials with high surface area and ultrafine particle sizes, such as nanodiamond or graphene. One plausible strategy to transform the powder form carbon to aerogel monolith is the self-assembly and hydrothermal method. It is reported that graphene oxide (GO), a graphene derivative, can be transferred into a 3D porous graphene monolith aerogel (Sun et al., 2020) via both the strong π-conjugated and Van der Waals interactions between their surface oxygen functional groups during the hydrothermal process (Sun et al., 2013; Mohandes and Salavati-Niasari, 2014). However, the graphene sheets are prone to restack into large blocks and thick films (Zhang et al., 2017), which seriously reduces the specific surface area and retards the electron transfer during the catalytic process. It’s well known that doping heteroatoms (eg. N, P, S, B) into graphene can effectively tune its electronic structure, thus influence the catalytic performance of graphene (Liu et al., 2019; Li et al., 2021), meanwhile doping with heteroatoms would also prevent graphene from restacking. Among numerous heteroatom systems, nitrogen element doping is one of the most mature technology, which is thoroughly investigated (Wu et al., 2017; Gupta et al., 2018; Jia et al., 2019; Liu et al., 2019) concerning the chemical structure, redox property and basic structure-function relations. N heteroatoms containing five valence electrons exhibits higher electronegativity than C atoms (3.04 vs. 2.55), which benefits activating the neighboring positively charged sp^2^-C and breaking the inertness of π electrons through electron transfer (Ding et al., 2013). Whereas, increasing the doping amount and the precise control of the chemical state of nitrogen are the main challenges for constructing N doped graphene materials (Wang et al., 2014).

Aiming at above scientific challenges, herein, we fabricated a series of porous nitrogen doped graphene monolith catalyst (NG) with GO and urea as precursors through self-assembly and hydrothermal method. During the hydrothermal process, the dopant urea containing -NH_2_ functional groups has strong interactions with the oxygen functional groups on GO, which effectively separates graphene layers from restacking and benefits nitrogen element to dope into the graphene. Meanwhile, the released NH_3_ during hydrothermal and pyrolysis process leads to the generation of rich hierarchical pores resulting in the enhancement of the surface area. The as-prepared NG aerogel monolith catalyst exhibits excellent thermal stability and high catalytic performance in the oxidative dehydrogenation reaction of propane. This work not only creates an efficient ODH nanocarbon catalyst but also addresses the problems regarding the powder-like nature of carbon materials, providing a new method to fabricate monolith carbon catalyst with enhanced heat and mass transfer ability, which has potential in practical heterogeneous catalysis.

## Experimental Section

### Synthesis of Graphene Oxide

The GO was prepared with a modified Hummers’ method (Marcano et al., 2010). Typically, 1.5 g of natural graphite, 180 ml of H_2_SO_4_ and 20 ml of H_3_PO_4_ were firstly mixed together at 50°C with magnetic stirring. Then 9 g of KMnO_4_ was slowly added with vigorous stirring for about 6 h. Finally, 100 ml of H_2_O_2_ (30 wt%) was dropped into above solution, and its color turned from dark brown to yellow with vigorous stirring for another 3 h. The suspension was centrifuged and washed with deionized water until the PH reaches 7. Finally, the GO dispersion in the concentration of 5 mg/ml was kept in 15°C for later use.

### Preparation of N-Doped Graphene Monolith

The N-doped graphene (NG) monolith was synthesized by the hydrothermal reduction of GO with urea as the N precursors and the following freeze-drying and further annealing process. Briefly, a certain amount of urea was dissolved in 120 ml of 5 mg/ml GO water dispersion, followed with vigorous stirring for about 20 min and the mixture was transferred into a 180 ml Teflon-lined autoclave maintaining at 180°C for 12 h. Then, the formed NG hydrogel was immersed in deionized water for 20 min to remove the residual agents. Next, the NG monolith was obtained by vacuum freeze-drying for 48 h. Finally, the NG monolith catalysts were treated at 550°C with the heating rate of 5°C/min under Argon atmosphere for 2 h. NG monolith were prepared with three different mass ratios of GO to urea (1:0.5, 1:1, 1:2) to control the doping amount of nitrogen in some extent, and the synthesized samples were denoted as NG-0.5 (1:0.5), NG-1 (1:1), NG-2 (1:2), respectively. For comparison, the reduced graphene oxide monolith without nitrogen doping was also prepared by the same method without introducing urea, which is denoted as G.

### Characterizations

Transmission electron microscopy (TEM) images of the catalysts were obtained by using FEI Tecnai T12 transmission electron microscope operating at 200 kV with a space resolution of 0.24 nm. The crystal structures were analyzed by XRD (Bruker D8 ADVANCE diffractometer, Cu-Kα λ = 1.5418 Å at a step scan of 2°min from 10° to 70°), Raman measurements were carried out with HORIBA LabRam HR800 Raman spectrometer equipped with the laser source at the wavelength of 532 nm, and the scanning electron microscopy (SEM) measurements were carried out with FEI Nano 450. The Brunauer-Emmett-Teller (BET) specific surface area (SSA) measurements were carried out at −196°C (ASAP3020). Thermogravimetric analysis was performed using NETZSCH STA 449 C with an air flow of 100 ml/min, and the catalysts were heated up to 900°C keeping for 20 min. XPS (X-ray photoelectron spectroscopy) analysis was carried out with ASCALAB 250 spectrometer equipped with a semispherical electron analyzer and Mg Kα (hν = 1253.6 eV) 300 W X-ray source. The binding energies were referenced to the C 1s line at 284.6 eV. The half-peak width of each oxygen or nitrogen species is controlled at 1.2–1.8 eV. The attenuated total reflectance FTIR measurements (ATR-IR) were recorded on a Thermo Nicolet iS10 ATR-FTIR system equipped with a liquid nitrogen-cooled MCT detector.

### Catalytic Test

The oxidative dehydrogenation reaction of propane was performed at 450°C in a fixed bed quartz tube reactor with 10 mm outside diameter. 40 mg catalyst was mounted into the reactor with certain amount of quartz wool as the cover and holder. The apparent activation energy test was carried out at 400–450°C with 40 mg catalyst. The reactant mixture containing 4% C_3_H_8_ and 2% O_2_ with He (99.999%) balance at a total flow rate of 15 ml/min and the space velocity is 2.25*10^4^ ml_gas_g_cat_^−1^h^−1^. The contact time is 4.44*10^−5^ g_cat_ h ml_gas_^−1^. The carbon balance for all the reactions were observed maintaining at 100 ± 5%. The kinetic analysis was performed under the kinetic region, where the propane conversion was controlled below 10% without the influence of diffusion limitation. All the reactants and products were analyzed by gas chromatography (Agilent 7890B) equipped with the flame ionization detector (FID) and thermal conductivity detector (TCD). The conversion of reactant (Con.), selectivity (S) and yield (Y) of product, formation rate of product (R_f_) and carbon balance (C_b_) were determined using Eqs 1–5, respectively. The letter C represents molarity of the products or reactants, with N and C_0_ representing the number of carbon atoms and the initial molarity of propane. The letter p, r, I, and m stand for product, reactant, summation notation and mass of catalyst, respectively.

$$Con.=\frac{\sum N_{p,i}C_{p,i}}{\sum N_{p,i}C_{p,i}+\sum N_{r,i}C_{r,i}}\times 100\%$$ (1)

$$S_{p,i}=\frac{N_{p,i}C_{p,i}}{Con.NC_{0}}\;\times 100\%$$ (2)

$$Y_{p,i}\left(\%\right)=Con.\;\times \;\;S_{p,i}$$ (3)

$$R_{f}\;\left(mmolg^{-1}h^{-1}\right)=\;\frac{Y_{p,i}\;\;\times C_{0}\;}{m_{catalyst}}$$ (4)

$$C_{b}\;=\frac{\sum N_{p,i}C_{p,i}+\sum N_{r,i}C_{r,i}}{NC_{0}}\;\times 100\%\;\;\;\;$$ (5)

## Results and Discussion

### Synthesis and Characterization of Graphene Monolith

**SCHEME 1:**
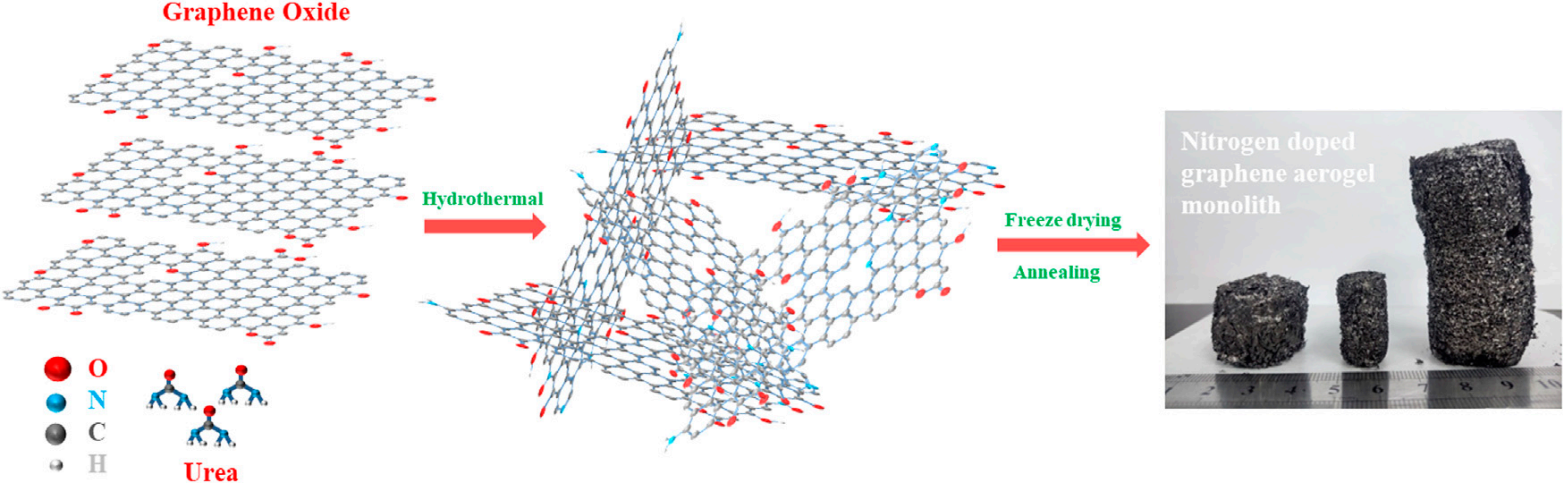
Schematic illustration of the fabrication process for the NG monolith

The fabrication process of the nitrogen doped graphene monolith is illustrated in Scheme 1. A certain amount of urea was highly dispersed into GO water dispersion under ultrasonic treatment, and then a homogeneous suspension of GO and urea could be obtained, which was chemically stable in the next few hours. Subsequently, the suspension was heated under high pressure via the hydrothermal treatment in which graphene oxide sheets were reduced and self-assembled through the strong π-conjugated and Van der Waals interactions between the functional groups to form a 3D graphene network with finely dispersed nitrogen element. During the hydrothermal process, urea plays a role of both the dopant and reducing agent because of its high nitrogen content. Next, 3D nitrogen doped graphene monolith was obtained after thoroughly washing with deionized water and the freeze-drying process. To remove the unstable component and improve the degree of graphitization, further annealing treatment at 550°C in Argon atmosphere was carried out. Finally, nitrogen doped graphene aerogel derived monoliths (NG) with different size in cylindrical shape could be obtained by using different types of the Teflon-lined autoclave as molds as shown in Scheme 1.

**FIGURE 1:**
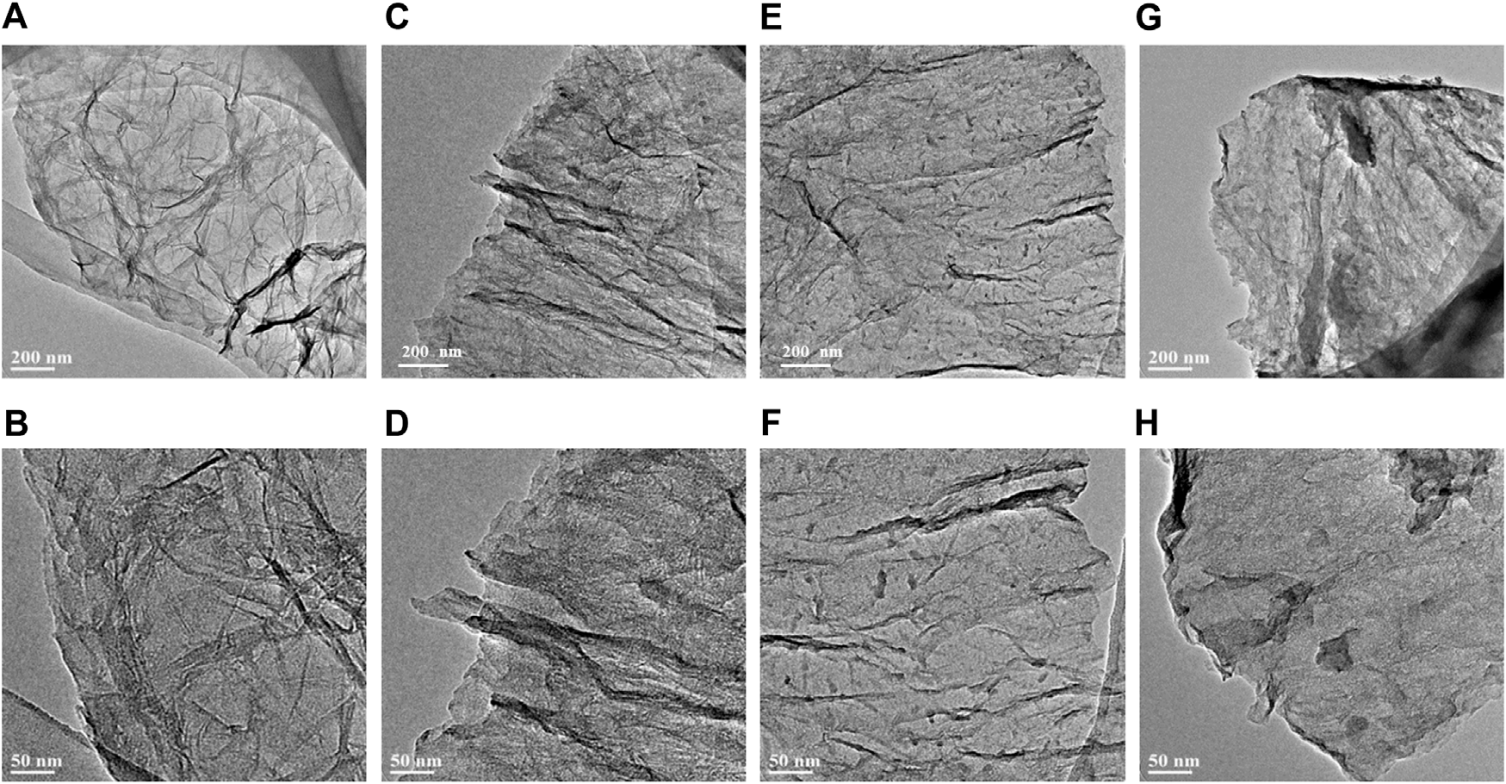
TEM images of G **(A,B)**, NG-0.5 **(C,D)**, NG-1 **(E,F)** and NG-2 **(G,H)**.

**FIGURE 2:**
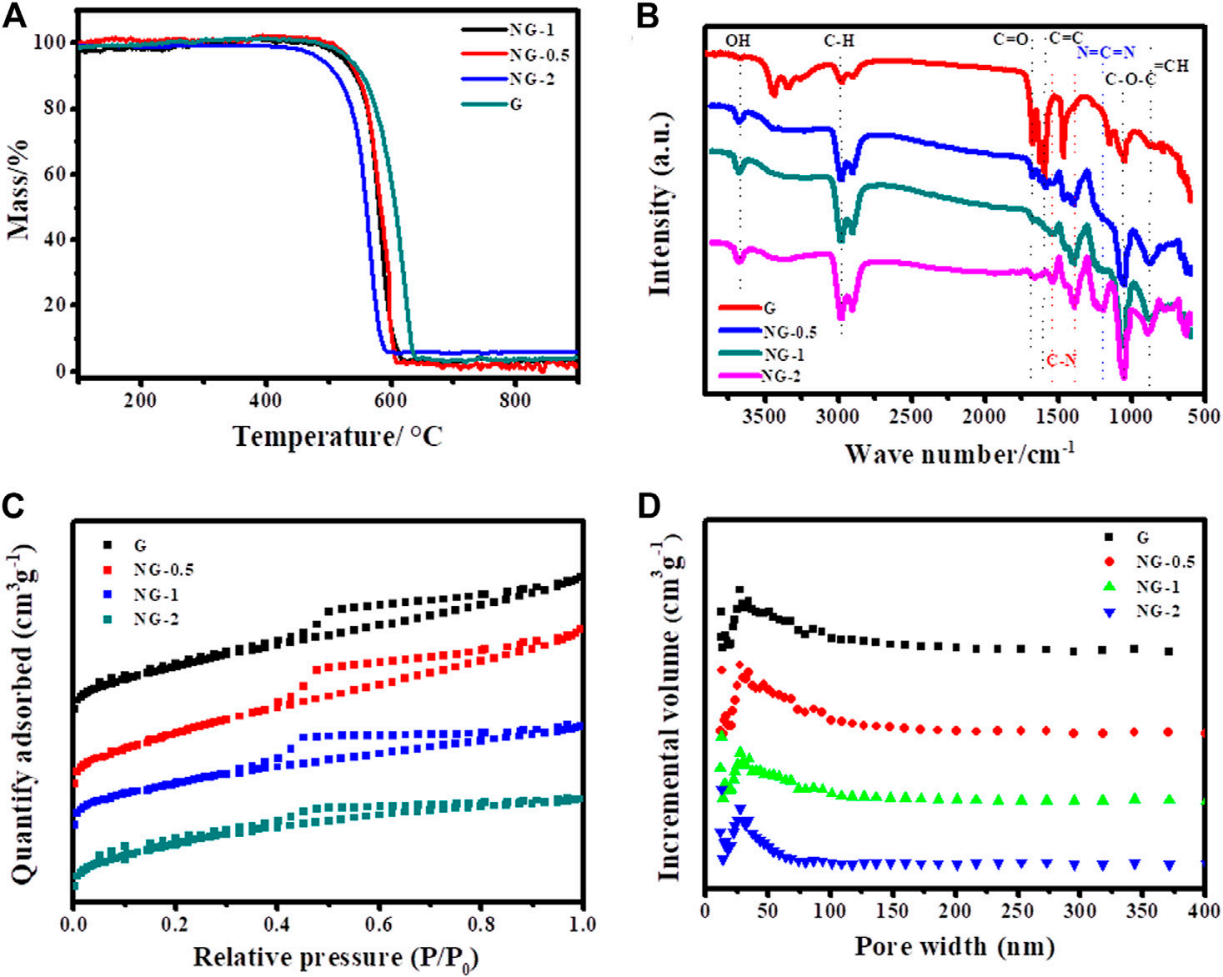
**(A)** TGA curves, **(B)** FTIR spectra, **(C)** Nitrogen adsorption–desorption isotherms and **(D)** Pore size distributions of G, NG-0.5, NG-1 and NG-2.

The overall morphology of the as-fabricated G, NG-0.5, NG-1 and NG-2 samples are firstly revealed by TEM as shown in Figure 1. Obvious wrinkle and folds appear in pure G, while the synthesized nitrogen doped graphene monolith samples NG-0.5, NG-1 and NG-2 show smooth surface, which should be resulted from the reduction effect of urea during the hydrothermal process. It can also be observed that small amount of amorphous carbon appear after nitrogen doping, and then it becomes more and more obvious with the increase of urea addition. It can be observed from the SEM images in Supplementary Figure S1 that both pure graphene and nitrogen doped graphene feature a 2D sheet like morphology, and the lateral sheet size is in the range of several and dozens of micrometers. The interconnected framework between the small sheets create a porous morphology, which benefits the improvement of the specific surface area. The thermogravimetric analysis (TGA) can characterize the thermal stability of samples. The TGA result (Figure 2A) shows that both of the pure graphene and nitrogen doped graphene exhibit relatively high thermal stability in the air atmosphere. The weight loss start after 500°C indicating that the as-fabricated graphene monolith catalysts maintain high thermal stability and can be applied in the oxidative dehydrogenation reaction of propane. The T_50_, which refers to the temperature at which the weight loss reaches 50% of the initial mass (Li et al., 2020), can be used to evaluate the thermal stability of the catalysts. The T_50_ values of NG-2、NG-0.5、NG-1 and G sample are determined at about 560、580、580 and 600°C, respectively, indicating that the thermal stability of the graphene aerogel exhibited slight decreases after nitrogen doping.

FTIR spectra are shown in Figure 2B, both the pure graphene and nitrogen doped graphene exhibit four typical peaks at approximately 1056, 1672, 2968 and 3670 cm^−1^, which could be assigned as the stretching vibration modes of C-O bond, stretching vibration modes of C=O bond, stretching vibration modes of C-H bond and stretching vibration modes of -OH bond, respectively (Weng, 2010). It is obvious that the signal of C=O functional group become weaker after nitrogen doping, and which is mainly because of the reducing effect of urea resulting in the decrease of oxygen amount. In addition, there are several obvious peaks in the nitrogen doped graphene aerogel at 1537, 1401 and 1201 cm^−1^, corresponding to the stretching vibration modes of C-N bond, the stretching vibration modes of C-N bond and the symmetrical stretching vibration modes C=N=C bond, respectively, which indicates that nitrogen element was indeed doped into graphene layer after the hydrothermal and calcination process (Wang et al., 2012; Lin and Su, 2014).

The porosity of the synthesized samples is investigated by N_2_ adsorption–desorption isotherms with the detailed information of specific surface area (SSA), pore size distribution (PSD) and pore volume. As shown in Figure 2C, all N_2_ adsorption–desorption profiles of NG-0.5, NG-1 and NG-2 exhibit a typical type IV isotherm in the medium to high pressure regions (p/p_0_ = 0.35–0.99), indicating the existence of rich mesoporous structures. In addition, two small unobvious steep peaks at low and high pressure (p/p_0_< 0.02, p/p_0_ > 0.96) can also be observed implying the presence of micropores and macropores (Zhao et al., 2015; Orooji et al., 2020). On the contrary, G sample exhibits indistinct adsorption hysteresis loop suggesting its less porous property than NG samples. The detailed pore structure information is presented in Supplementary Table S1. The SSA for G, NG-0.5, NG-1 and NG-2 are calculated at about 322, 421, 369 and 521 m^2^g^−1^ with corresponding total pore volume up to 0.30, 0.37, 0.27 and 0.36 cm^3^g^−1^, respectively. The increase of the SSA indicates the pillared effect of urea via impeding the restacking of graphene sheets, and the pore size distribution curve ((Figure 2D)) shows that the pores in these four samples are mainly in mesoporous nature. The XRD diffraction patterns (Supplementary Figure S2A) shows the crystal structures of the aerogels. It can be observed that four obvious diffraction peaks appear in the four samples at 24.7° (G), 24.7° (NG-0.5), 24.6° (NG-1) and 24.4° (NG-2) with corresponding d-space of 0.360, 0.362, 0.373 and 0.380 nm, which could be indexed to the (0 0 2) inter-plane distance of graphene based on the Bragg’s equation (Saleem et al., 2018). All samples exhibit similar diffraction peaks indicating the aerogel monolith keep intact structure of graphene after nitrogen doping.

Raman spectroscopy is another powerful method for characterizing the defect degree of N-doped graphene monolith (Yan P. Q. et al., 2019). Supplementary Figure S2B is the Raman spectrum of NG and G samples, in which there are two distinct peaks at approximately 1350 and 1580 cm^−1^, corresponding to the D and G peaks of graphene, respectively (Yan P. Q. et al., 2019). In general, the value of I_D_/I_G_ represents the defect degree of carbon materials, the higher the value the more defects existing in nanocarbon materials. The pure graphene aerogel exhibits a relatively high I_D_/I_G_ value of 2.07 indicating that there are many defects in the graphene monolith ascribing to strong oxidation effect of concentrated sulfuric acid and potassium permanganate in the modified Hummers’ produce process. The I_D_/I_G_ value drop after a certain amount urea addition because of the reduction effect of urea and then increase slightly with the addiction amount augment. For the nitrogen doped graphene monolith samples, the I_D_/I_G_ value increased slightly from 1.93 to 2.15 mainly because nitrogen atoms enter the graphite lattice inducing the hexagonal distortion and increasing the lattice space thus lead to the defect degree increase. In addition, micro-holes caused by ammonia release during hydrothermal process also contribute to the increase of structure defects (Cao et al., 2018).

**FIGURE 3:**
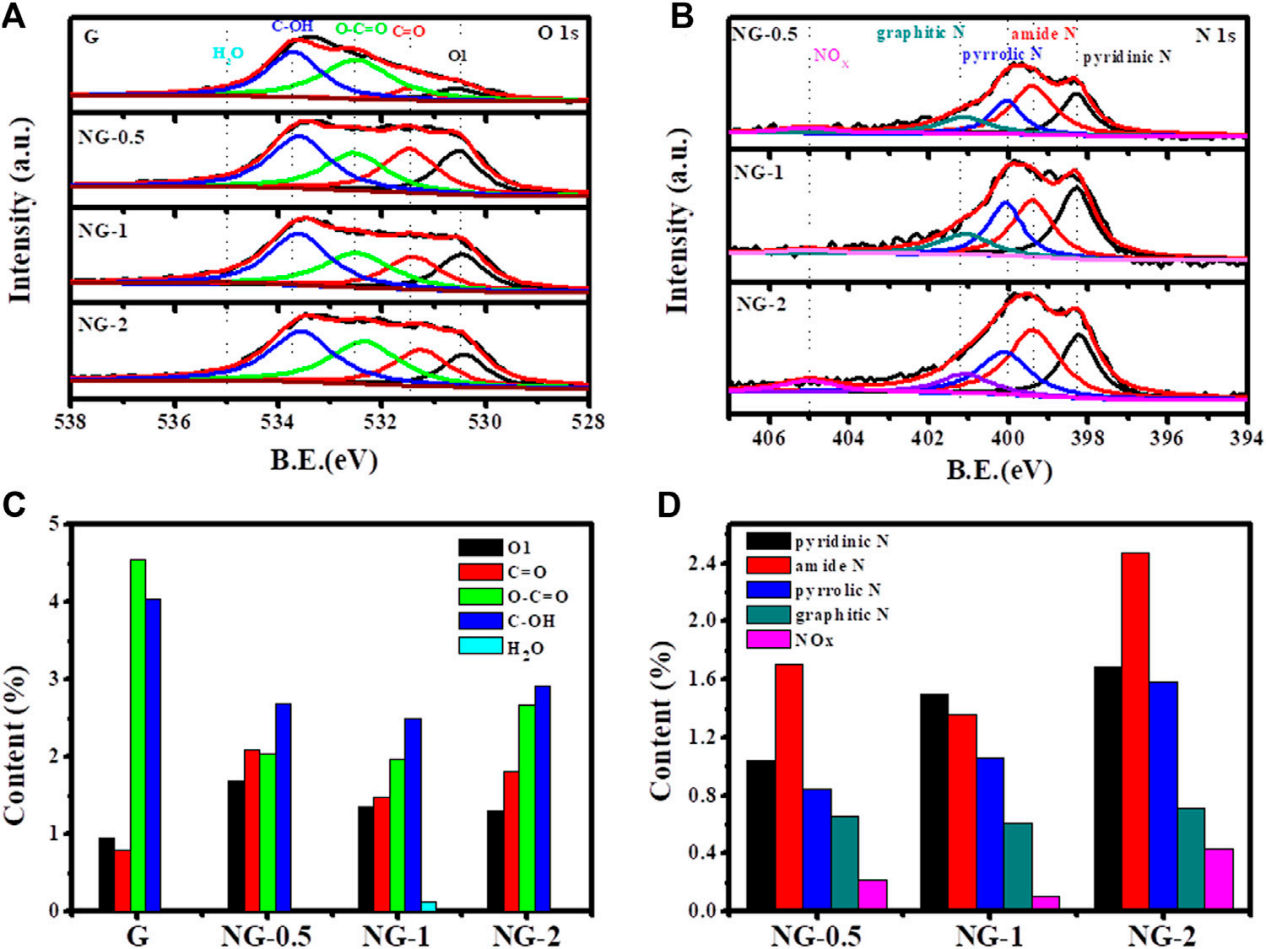
High resolution O 1s **(A)** and N 1s **(B)** XPS of graphene monolith; content of different O species **(C)** and N species **(D)** in graphene monolith samples from XPS and corresponding deconvolution results.

The XPS analysis is further used to verify the elemental composition, contents and chemical environment in the G and NG samples. As shown in Supplementary Figure S3, two obvious peaks locate at around 285 and 532 eV corresponding to C 1s and O 1s in the XPS survey scan spectrum of the above samples indicates the coexistence of C and O element in both pure and nitrogen doped graphene aerogel. The N 1s peak at 399 eV in those three nitrogen doped samples verifies that the nitrogen element is indeed doped into the graphene, which is consistent with the FTIR results. No metal element peak in the survey spectrum is observed, indicating that no metal or metal oxide impurities appear on the surface of the graphene materials. The amount of oxygen element in the pure graphene is 10.3 at % based on XPS result, and the value decreases to about 8.0 at % after nitrogen doping(shown in Supplementary Table S2), mainly because of the reduction effect of urea. O1s and N1s spectra are further fitted by Gaussian and Lorentz peaks to determine the chemical states of the O and N elements (Liu et al., 2017; Li et al., 2020). The O1s XPS fine spectrum of each sample is deconvoluted (shown in Figure 3A), and the integrated peak area is obtained, representing the relative content ratio of each species. The peaks at 530.7 ± 0.1 eV, 531.4 ± 0.1 eV, 532.3 ± 0.1 eV, 533.5 ± 0.1 eV and 535 ± 0.1 eV corresponds to the highly conjugated quinone C=O (O1), ketone C=O (O2), carboxyl COOH (O3), hydroxyl groups C-OH (O4) and H_2_O, respectively. The detailed deconvolution results (shown in Table 1) indicate that the quinone C=O (O1) and ketone C=O (O2) amount increase obviously from 0.95 at % to 1.35 at % and 0.79 at % to 1.47 at % after nitrogen doping. It is reported that ketone C=O and quinone C=O plays a key role in alkane dehydrogenation reactions (Guo et al., 2017), and this increasing amount of active oxygen species may enhance the catalytic performance. The nitrogen contents of NG 0.5, NG-1 and NG-2 sample are 4.46 at %, 4.62 at % and 6.88 at %, respectively, increasing with the addition amount of urea precursors. We divide the N1s XPS fine spectra into five different peaks (shown in Figure 3B), namely 398.4 ± 0.1 eV for pyridinic N specie, 399.4 ± 0.1 eV for amide N, 400.1 ± 0.1 eV for pyrrolic N, 401.1 ± 0.1 eV for graphitic N and 405 ± 0.1 eV of N-oxide (Arrigo et al., 2010). Among these three nitrogen doped graphene monolith samples, the NG-2 exhibit the highest graphitic N content of 0.71 at %, higher than 0.66 at % and 0.61 at % in the other two samples. Chen et al. (2013) supposed that graphitic N species is a critical factor to facilitate the catalytic performance of nitrogen doped nanocarbon. The Auger electron spectrum (AES) is another tool to characterize the electron transfer between different elements. Because Auger processes involve three electrons, their chemical shifts tend to be much more sensitive than those of XPS (Seah, 1984). The AES results (Supplementary Figure S4) of these four samples including G and NG exhibit that the O KLL peak of pure graphene located at 510.3 eV, and the peak shifts to 511.2, 512.1 and 511.1 eV for NG-0.5, NG-1 and NG-2, respectively (shown in Supplementary Table S3). The red shift trend (Lee et al., 2018) after nitrogen doping indicates the electron transferred from nitrogen element to oxygen element. Previously reported results (Hu et al., 2010; Ni et al., 2012; Chen et al., 2013) suggest that the density of π electron cloud on the unsaturated double bond of the olefin is relatively high, and the repulsion between olefin and nitrogen-doped graphene with rich electron density is stronger than that of pure graphene, which is more conducive to desorption of the product propylene on the catalyst surface, avoiding the deep oxidation of propylene and improving the selectivity of alkane ODH reactions. Therefore, doping nitrogen element to graphene may facilitate the catalytic performance in ODH reactions.

**TABLE 1:** Content of different O and N species in graphene monolith samples from XPS and corresponding deconvolution results.

Sample	O1	C=O	O-C=O	C-OH	H_2_O	pyridinic N	amide N	pyrrolic N	graphitic N	NOx
G	0.95	0.79	4.55	4.03	0.00	—	—	—	—	—
NG-0.5	1.68	2.08	2.04	2.69	0.00	1.04	1.70	0.84	0.66	0.22
NG-1	1.35	1.47	1.96	2.49	0.12	1.50	1.36	1.06	0.61	0.10
NG-2	1.30	1.81	2.67	2.92	0.00	1.69	2.47	1.58	0.71	0.43

### Catalytic Performance

**FIGURE 4:**
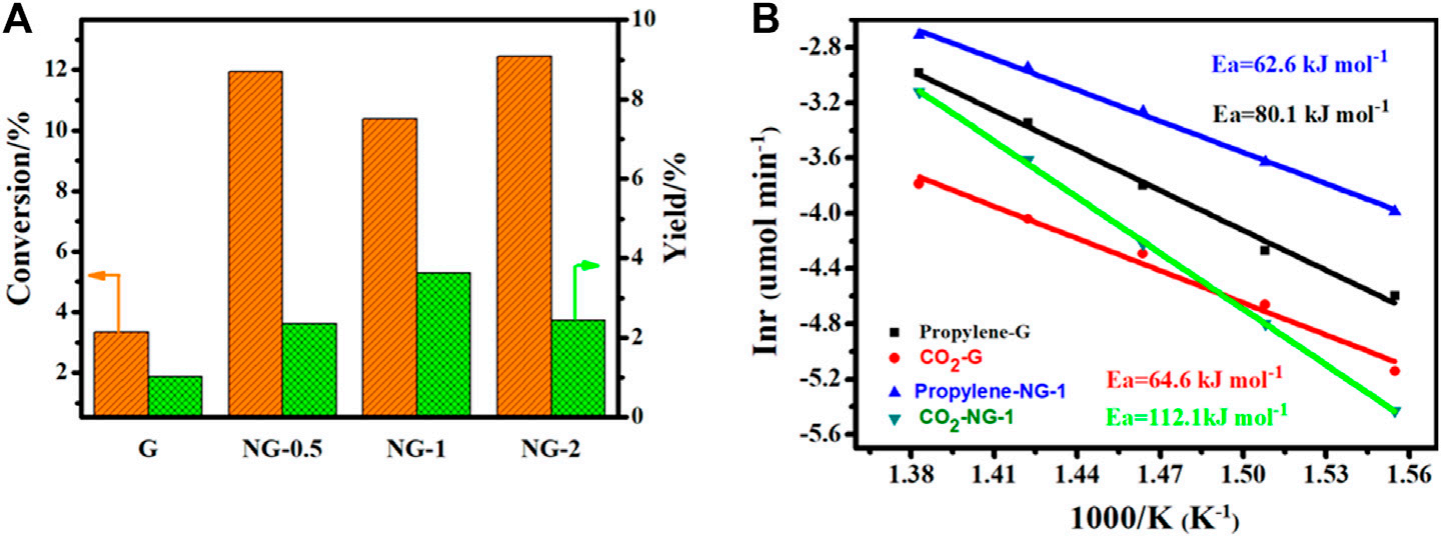
**(A)** Conversion of propene and propylene yield for different catalysts in ODH reaction. **(B)** Arrhenius plots and apparent activation energy for G and NG-1. (reaction conditions: 40 mg catalysts, 4% propane, ambient pressure, 15 ml/min, He balance, 400–450°C.

The catalytic performance of G, NG-0.5, NG-1 and NG-2 is explored with oxidation dehydrogenation reaction of propane, and there is no reaction activity could be observed in the control blank experiment (without catalyst at 450°C as shown in Supplementary Figure S5A). As shown in Figure 4A, the yield of propylene for G, NG-0.5, NG-1 and NG-2 catalyst is observed at 1.9, 3.6, 5.3 and 3.7%, respectively. Obviously, the synthesized nitrogen doped graphene monolith catalysts exhibit higher catalytic activity in ODH than pure graphene monoliths. The NG-1 sample shows the best catalytic performance with initial conversion up to 10% at 450°C, and the selectivity to propylene is over 55%, with the main by-product being COx (as shown in Supplementary Figure S5B). Both the pure G and NG samples exhibit certain catalytic activity, but the NG-1 monolith catalyst exhibits over twice higher activity than pure graphene monolith (propylene yield at 5.3 vs. 1.9%). The catalytic activity of these monolith materials can last over 10 h without obvious deactivation under the chosen reaction conditions (450°C, 4% of propane, 2% of O_2_). Comparing with metal oxide catalysts (as shown in Supplementary Table S4), the propane conversion of nitrogen doped graphene monolith shows a decent propane conversion but a relatively high selectivity of propylene, (Blasco and Nieto, 1997; Argyle et al., 2002; Carrero et al., 2014), which may be due to its weaker acidity than metal oxide catalysts. Comparing with metal-free catalyst, such as carbon nanotube (CNT) and nanodiamond (ND) etc., the NG monolith exhibits relatively high catalytic activity in the oxidative dehydrogenation of propane and is a very promising metal free catalyst.

The apparent activation energy (Ea) for ODH on NG-1 is determined at 62.6 ± 1.9 kJ mol^−1^ based on the formation rate of propylene (as shown in Figure 4B), which is obviously lower than that on pure graphene (80.1 ± 3.4 kJ mol^−^) under the same reaction conditions. The promotion effect is mainly because of the increase amount of the active sites, namely the surface content of ketone C=O and quinone C=O (O1) species on NG surface after the introduction of nitrogen species. Especially the quinone C=O groups, which is commonly regarded as the active sites for propane ODH reactions. At the same time, the apparent activation energy (Ea) for CO_2_ formation on NG-1, calculated based on the formation rate of CO_2_, is much larger than that of pure graphene (112.1 ± 1.2 kJ/mol vs. 64.6 ± 4.3 kJ/mol) indicating that it’s more difficult to produce CO_2_ than propylene for NG-1 comparing to that for pure graphene catalyst in ODH reaction. This finding also explains why the active NG-1 has shown higher propylene selectivity in the presence of oxygen at high temperature.

It is reported (Xu and Li, 2004; Zhang et al., 2007; Liang et al., 2009; Seredych and Bandosz, 2009) that structure defects and nitrogen content are two main key factors affecting the catalytic performance of nanocarbon catalysts in alkane ODH reactions. Previous studies (Xu and Li, 2004) have shown that the adsorption and dissociation of oxygen molecule reactants mainly occurs on the defect sites on carbon catalysts, because the charge density of the carbon atom here is higher than that of carbon skeleton of the graphene layer, and the electrons are easily transferred from the carbon atom to the adsorbed oxygen molecule. For the nitrogen doped graphene catalyst, although the doped nitrogen atoms entering the graphitic lattice caused the distortion of carbon skeleton and increased the lattice space, the reduction effect of urea during the hydrothermal process reduced the oxygen content and structure disorder thus leading to the structure defect decrease. As a result of these two opposite effects, the defect degree dropped after a certain amount of urea addition (NG-0.5) and then increased slightly with the addiction amount augment (NG-1 and NG-2) as shown in the Raman spectrum result. However, the structure defect degree of NG changed only slightly with the increase of nitrogen doping (the I_D_/I_G_ value varying from 1.93 to 2.15), which means that the slight alternation of the structure defect degree may be not the main reason for the catalytic performance enhancement, although it may contribute to the catalytic performance improvement in some degree. On the other hand, some experimental studies and related theoretical calculation results (Chen et al., 2013) (Hu et al., 2010; Shan and Cho, 2010) have shown that the graphitic nitrogen benefits the catalytic performance enhancement. Chen et al. (2013) correlated the content of graphite nitrogen with activation energy and obtained a linear equation for quantifying the effect of nitrogen doping on the decrease of the apparent activation energy. For our synthesized nitrogen doped graphene monolith (NG samples), the increasing amount of ketone C=O and quinone C=O and the introduction of graphitic nitrogen should be the key factors for enhancing the catalytic performance of carbon materials.

## Conclusion

In summary, we have designed a new synthetic route to fabricate nitrogen doped graphene-based monolithic carbon catalysts via the sequent self-assembly, hydrothermal and post thermo-treatment procedures with urea and graphene oxide as precursors. During the hydrothermal process, amino functional groups in urea interact with the oxygen functional groups in graphene resulting in a slight decrease of total oxygen content but an increase of quinone C=O (O1) and ketone C=O amount. The FTIR and XPS characterizations indicate that the nitrogen element indeed doped into the graphene aerogel and the surface nitrogen content is up to 6.88 at % (NG-2). The catalytic performances of these samples are evaluated in oxidative dehydrogenation of propane reaction. The homogeneously distributed nitrogen species effectively improve the yield of propylene (5.3% vs. 1.9%) and lower the activation energy of propane ODH reactions (62.6 kJ mol^−1^ vs. 80.1 kJ mol^−1^). The optimized doping amount at 1:1 weight content of the graphene to urea precursors exhibits the best promotion effect of the catalyst. The present work paves the way for developing nitrogen doped graphene monolith catalysts for ODH reactions with high efficiency. The detailed reaction mechanism for the promotion effect and the basic structure–function relations for these series of carbon catalysts still need further exploration.

## Data Availability

The original contributions presented in the study are included in the article/Supplementary Material, further inquiries can be directed to the corresponding author.
