# Supplementary material for: Nitrogen-Doped Graphene Monolith Catalysts for Oxidative Dehydrogenation of Propane
Source: Front Chem. 2021 Oct 15;9:759936. doi: 10.3389/fchem.2021.759936 (PMC8554143; doi:10.3389/fchem.2021.759936)
Supplement: Supplementary file 1 [file DataSheet1.pdf]

Supplementary Materials

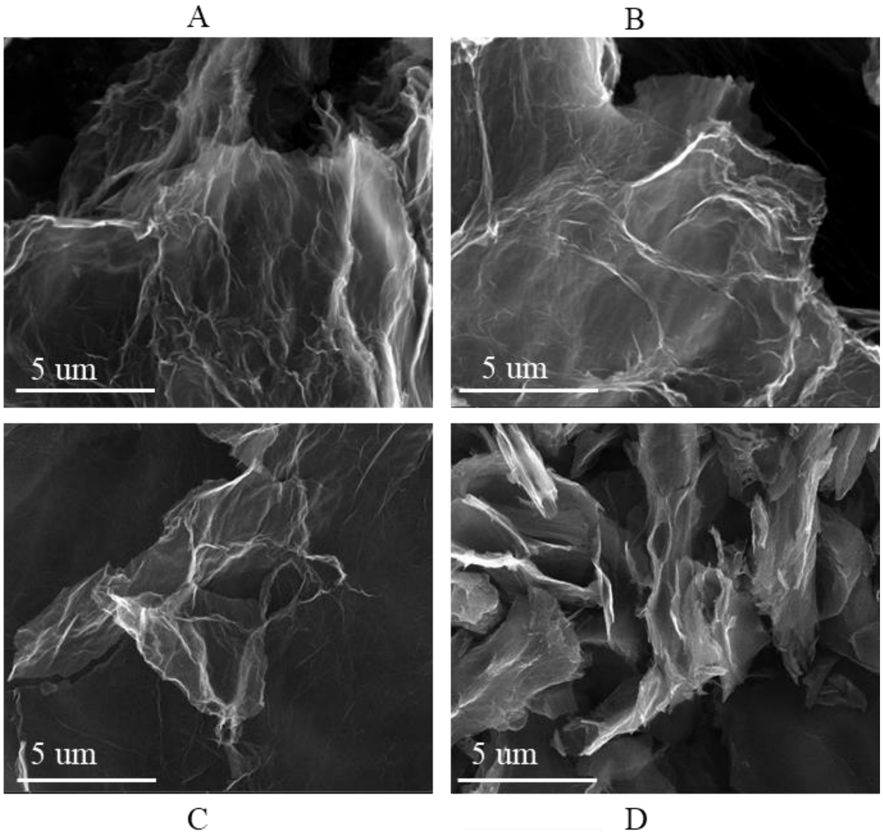

Figure S1 | SEM images of G (A), NG-0.5 (B), NG-1 (C) and NG-2 (D).

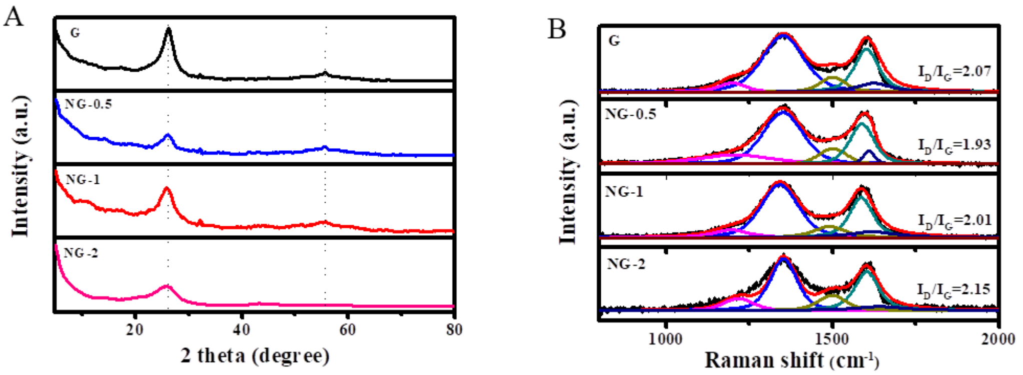

Figure S2 | (A) XRD patterns of G, NG-0.5, NG-1 and NG-2. (B) Raman spectra of G, NG-0.5, NG-1 and NG-2.

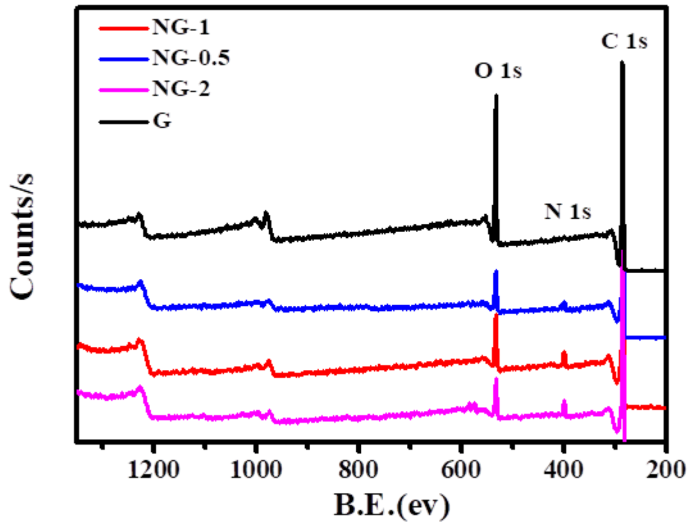

Figure S3 | XPS survey spectra of G, NG-0.5, NG-1 and NG-2.

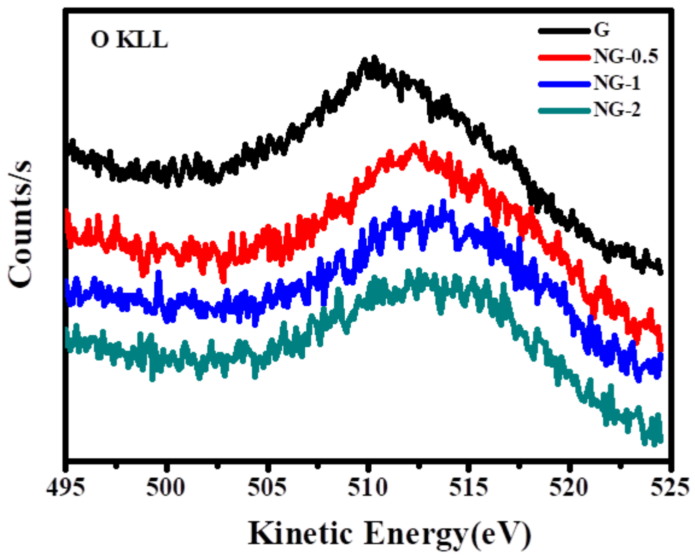

Figure S4 | Kinetic Energy of O KLL in G, NG-0.5, NG-1 and NG-2.

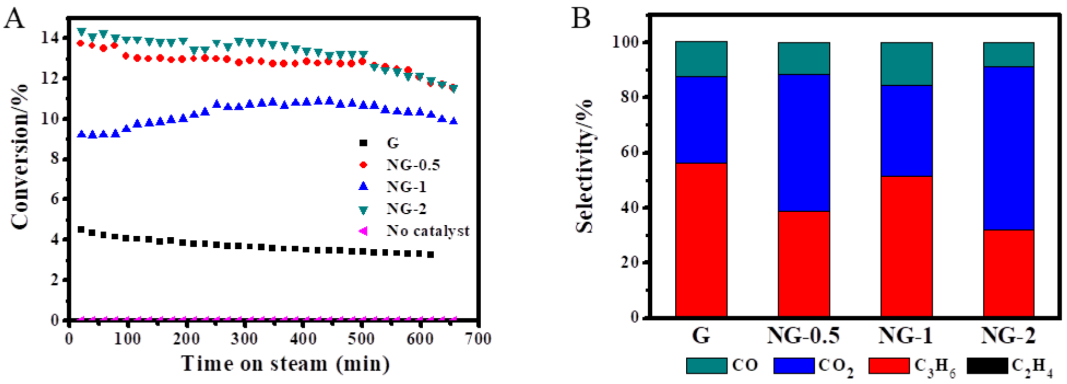

Figure S5 | (A) Propane conversion as a function of reaction time. (B) Product selectivity of different catalysts in ODH reaction. (reaction condition: 40 mg catalysts, 4% propane, 2% O_2, ambient pressure, 15 ml/min, He balance, 450 °C)

Table S1 | Specific surface area, pore volumes and average pore sizes of G, NG-0.5, NG-1 and NG-2.

Sample	SSA (m^2g^{-1})	Pore volume (cm^3g^{-1})	$V_{meso/total}$ (%)	Pore size (nm)
G	322	0.30	99.9	3.7
NG-0.5	421	0.37	94.59	3.5
NG-1	369	0.27	77.78	2.9
NG-2	521	0.36	72.22	2.8

Table S2 | Atomic percentage of C, O and N elements in graphene monolith.

Sample	**C (at.%)**	**O (at.%)**	**N (at.%)**
G	90	10	----
NG-0.5	87	8	4
NG-1	88	7	5
NG-2	84	9	7

Table S3 | Auger peak position of G, NG-0.5, NG-1 and NG-2.

Sample	G	NG-0.5	NG-1	NG-2
Auger peak (eV)	510.3	511.2	512.1	511.1

Table S4 | Comparison of the catalytic performance for some typical catalysts in propane ODH reactions

Catalyst	Temperature (℃)	C_3H_8 conversion (%)	Selectivity (%)			Reference
			C_3H_6	CO	CO_2	
5VZr	400	13.8	38.7	39.9	21.4	(Christodoulakis et al., 2004)
5VTi	400	27.1	17.5	61.0	21.5	(Christodoulakis et al., 2004)
VOx/Al_2O_3	400	4.6	83	--	--	(Frank et al., 2007)
VOx/Al_2O_3	500	15	61	--	--	(Frank et al., 2007)
85%NiO-POM	450	72	20	0	80	(Zhang et al., 2009)
70%NIO-POM	450	11	75	5.9	16	(Zhang et al., 2009)
$NiMoO_4$	560	27	59.3	--	--	(Stern and Grasselli, 1997)
V-Ti/SBA-15	500	6.7	51	--	--	(Carrero et al., 2014)
CMK-3	450	11	55	--	--	(Michorczyk et al., 2012)
ND	450	4.7	63	--	--	(Sun et al., 2015)
CNT	460	7.1	22	--	--	(Frank et al., 2009)
CNT	460	14.9	12	--	--	(Frank et al., 2009)
h-BN	490	5.4	85	--	--	(Grant et al., 2016)
BNNT	490	6.5	78	2	3	(Grant et al., 2016)
NG monolith	450	12	60	13	27	This work

Reference

Christodoulakis, A., Machli, M., Lemonidou, A.A., and Boghosian, S. (2004). Molecular structure and reactivity of vanadia-based catalysts for propane oxidative dehydrogenation studied by in situ Raman spectroscopy and catalytic activity measurements. *Journal of Catalysis* 222(2)**,** 293-306. doi: 10.1016/j.jcat.2003.10.007.

Frank, B., Dinse, A., Ovsitser, O., Kondratenko, E.V., and Schomäcker, R. (2007). Mass and heat transfer effects on the oxidative dehydrogenation of propane (ODP) over a low loaded VOx/Al2O3 catalyst. *Applied Catalysis A: General* 323**,** 66-76. doi: 10.1016/j.apcata.2007.02.006.

Zhang, Q., Cao, C., Xu, T., Sun, M., Zhang, J., Wang, Y., et al. (2009). NiO–polyoxometalate nanocomposites as efficient catalysts for the oxidative dehydrogenation of propane and isobutane. *Chemical Communications* (17)**,** 2376-2378. doi: 10.1039/B823369A.

Stern, D.L., and Grasselli, R.K. (1997). Propane Oxydehydrogenation over Molybdate-Based Catalysts. *Journal of Catalysis* 167(2)**,** 550-559. doi: 10.1006/jcat.1997.1568.

Carrero, C., Kauer, M., Dinse, A., Wolfram, T., Hamilton, N., Trunschke, A., et al. (2014). High performance (VOx)n–(TiOx)m/SBA-15 catalysts for the oxidative dehydrogenation of propane. *Catalysis Science & Technology* 4(3)**,** 786-794. doi: 10.1039/C3CY00625E.

Michorczyk, P., Kuśtrowski, P., Niebrzydowska, P., and Wach, A. (2012). Catalytic performance of sucrose-derived CMK-3 in oxidative dehydrogenation of propane to propene. *Applied Catalysis A: General* 445-446**,** 321-328. doi: 10.1016/j.apcata.2012.08.044.

Sun, X., Ding, Y., Zhang, B., Huang, R., and Su, D.S. (2015). New insights into the oxidative dehydrogenation of propane on borate-modified nanodiamond. *Chemical Communications* 51(44)**,** 9145-9148. doi: 10.1039/C5CC00588D.

Frank, B., Zhang, J., Blume, R., Schloegl, R., and Su, D.S. (2009). Heteroatoms Increase the Selectivity in Oxidative Dehydrogenation Reactions on Nanocarbons. *Angewandte Chemie-International Edition* 48(37)**,** 6913-6917. doi: 10.1002/anie.200901826.

Grant, J.T., Carrero, C.A., Goeltl, F., Venegas, J., Mueller, P., Burt, S.P., et al. (2016). Selective oxidative dehydrogenation of propane to propene using boron nitride catalysts. *Science* 354(6319)**,** 1570. doi: 10.1126/science.aaf7885.
